# Nurse Practitioners' Experiences of Transitioning to and Working in the Pioneering Nursing Role: An Interview Study

**DOI:** 10.1111/jan.16560

**Published:** 2024-10-23

**Authors:** Birgitta Ljungbeck, Elisabeth Carlson, Katarina Sjögren Forss

**Affiliations:** ^1^ Department of Care Science, Faculty of Health and Society Malmö University Malmö Sweden; ^2^ Municipal Healthcare in Hässleholm Management of Care and Welfare Hässleholm Sweden

**Keywords:** experiences, interviews, nurse practitioners, qualitative, transition

## Abstract

**Background:**

Nurse practitioners (NPs) play a crucial role in healthcare by providing person‐centred and high‐quality care. Their contributions encompass enhanced accessibility to healthcare and reduced hospital admissions and cost‐effectiveness. They usually hold a master's degree in nursing, incorporating expanded clinical skills to manage patients in different settings and address both nursing and medical needs. Despite the global development of the role, its implementation varies, presenting challenges related to role clarity, regulation and acceptance by other healthcare professionals.

**Aim:**

To explore how nurses who are or have been employed as NPs describe their experiences of transitioning to and working in the NP role in Sweden.

**Design:**

A qualitative interview study.

**Method:**

This study explores the experiences of 15 NPs in Sweden, using a snowball sampling strategy and semi‐structured interviews. The data were collected through interviews in April and May 2022 and analysed with inductive content analysis.

**Findings:**

The present study explores the experiences of NPs as they transition from experienced nurses to NPs, emphasising motivations, educational challenges, acceptance and autonomy. Transitioning into the NP role initially brought isolation that evolved into acceptance. They recognised their strength in integrating medical and nursing competencies for holistic care. Leadership was pivotal, with managerial support crucial for the successful implementation of the role. Autonomy‐related challenges, such as prescription rights and dependency on physicians, underscored the need for a protected professional title and national guidelines.

**Conclusion:**

This study enriches the evolution of the NP role in Sweden, offering vital insights into the ongoing national‐level dialogues on NP role development in Sweden.

**Impact:**

The paper's relevance extends globally by providing valuable perspectives on NPs practicalities and fostering international understanding and advancement.

**Reporting Method:**

Standards for reporting qualitative research.

**Patient or Public Contribution:**

Not applicable.

**Summary**

- Decision‐makers must understand the nurse practitioner role to develop effective national guidelines.
- The lack of national guidelines regulating the nurse practitioner role limits their autonomy in practice.
- Nurse practitioners face challenges entering the role without managerial support.

## 1 Introduction

Countries around the world are grappling with the challenges of recruiting and retaining healthcare providers, given the persistent shortage of such professionals. The nurse practitioner (NP) role has emerged as one crucial solution to address these issues (Fougère et al. 2018; Jean et al. 2019). The NP role originated in the USA during the 1960s to enhance healthcare accessibility and has since been adopted by an increasing number of countries (Delamaire and Lafortune 2010). NPs are highly competent nurses providing high quality, person‐centred care and promote patient safety; thus, there is growing global interest in developing the NP role (Jean et al. 2019; Kippenbrock et al. 2019). Evaluations confirm that integrating the NP role into healthcare teams enhances accessibility to healthcare (Kippenbrock et al. 2019), reduces hospital admissions and readmissions (Buerhaus et al. 2018) and indicates cost‐effectiveness (Liu et al. 2020).

## 2 Background

NPs are minimally educated at the master's level, in alignment with the recommendation of the International Council of Nurses (Schober et al. 2020). The European Federation of Nurses Associations (2023) has delineated a workforce matrix categorising nursing levels in Europe: the NP role is positioned as the third tier of nursing; level two is the specialist nurse, and level one is the registered nurse (European Federation of Nurses Associations 2023). NPs' unique competence in advanced nursing and extended medical skills enables them to work autonomously in clinical practice, effectively managing both nursing and medical needs. The result is a high continuity of healthcare providers, fostering conditions to provide genuinely person‐centred care (Morilla‐Herrera et al. 2016).

Despite the positive effects noted, the implementation of the NP role within healthcare teams is complex and uneven, often stemming from inadequate preparatory groundwork (Fougère et al. 2018; Henni et al. 2019). Insufficient role clarity, along with ambiguous mandates and regulations to clarify the NPs scope of practice and level of autonomy, creates confusion, leading to clashes within those teams, whose members may be unsure of the NP's function (Henni et al. 2019; Torrens et al. 2020). The absence of role clarity also contributes to apprehension among other nurses and physicians, who may perceive NPs as a threat (Faraz 2016; Torrens et al. 2020). A Norwegian study revealed that other nurses expressed concerns about potential displacement from duties for which they are qualified in favour of NPs (Boman, Egilsdottir Ösp, and Fagerström 2018). Physicians' reluctance to accept the NP role reflects doubts over NP competence and a fear of losing power within the existing hierarchical structure. Consequently, negative attitudes from physicians towards NPs can impact collaboration, limiting NPs' ability to work to their fullest capacity (Faraz 2016; Henni et al. 2018). This is particularly evident when the NP role is implemented without adequate preparation, and confusion arises regarding whether NPs belong to the nursing or medical disciplines. It is therefore essential to underscore that the NP role is not intended to replace physicians but rather to complement them, leveraging the unique competence of NPs to benefit both patients and colleagues (Chavez, Andrew, and Ramelet 2018; Torrens et al. 2020). For instance, Chavez, Andrew, and Ramelet (2018) illustrate how the autonomous and collaborative NP role addresses gaps in healthcare delivery, leading to enhanced outcomes and patient satisfaction. Clarifying this distinction becomes crucial in addressing challenges related to role ambiguity and potential resistance from other healthcare professionals. Furthermore, it is imperative to address regulatory barriers, especially those concerning prescription rights and other responsibilities traditionally held by physicians, such as authorising blood samples, X‐rays and other investigations. Uncertainty regarding these matters affects the scope of practice for NPs and contributes to varying levels of autonomy among NPs in different contexts (Chavez, Andrew, and Ramelet 2018; Torrens et al. 2020).

In Sweden, efforts are underway to establish a cohesive framework for developing the NP role, driven by national interest and ongoing healthcare reform aimed at advancing person‐centred care (The National Health Competence Council 2023). However, the NP role currently lacks official recognition (Ljungbeck, Carlson, and Sjögren‐Forss 2023). Unlike registered nurses and specialist nurses, the Swedish educational system has not yet adopted the NP role as a regulated role or protected professional title. Consequently, the scope of practice and the level of autonomy for NPs are not yet defined (Ljungbeck, Carlson, and Sjögren‐Forss 2023). However, the NP role is considered a third tier of nursing, requiring a two‐year master's degree according to a proposal by The Swedish Society of Nursing, the Swedish Association of Health Professionals (2021). Registered nurses hold a bachelor's degree, while specialist nurses earn 60–75 credits in advanced‐level courses to obtain a one‐year master's degree (Råholm et al. 2010). Furthermore, understanding prescription rights for nurses in Sweden is crucial for NP role development; limitations on prescribing impact their autonomy and scope of practice (Altersved et al. 2011; Lindblad et al. 2010). Upon completing a pharmacology course, registered nurses can prescribe certain medications from a limited list if they are employed in county council, primary care including home healthcare and elderly care. No extended prescription right for NPs currently exist in the country (Maier 2019).

Despite extensive international research on the NP role, its specific profile in the Swedish context has received limited attention. However, recent Swedish studies echo international findings that highlight NPs' potential to enhance healthcare access, deliver person‐centred care and support healthcare teams. These studies also shed light on regulatory obstacles, particularly responsibilities traditionally held by physicians, underscoring the need for enhanced NP role regulation (Ljungbeck, Carlson, and Sjögren‐Forss 2022, 2023). Although NP role development is in its early stages, some local initiatives have been undertaken without national endorsement. Some of them have not met their goals and have been discontinued, while others have been successful in integrating the NP role effectively. In collaboration with local universities and healthcare organisations, the NP role has been implemented in surgical and emergency departments at a few hospitals in central Sweden and in primary care at some healthcare centres in northern Sweden (Altersved et al. 2011; Andregård and Jangland 2015; Lindblad et al. 2010). Nonetheless, there remains a scarcity of critical research evaluating these local endeavours. Therefore, it is valuable to further evaluate these local initiatives as insights from these can enrich broader national‐level dialogues by reflecting NPs' experiences in transitioning to and working in this role.

## 3 The Study

### 3.1 Aim

The present study aims to explore how nurses who are or have been employed as NPs describe their experiences of transitioning to and working in the NP role in Sweden.

### 3.2 Design

A qualitative descriptive research design using individual semi‐structured interviews was used for this study (Polit and Beck 2020) and analysed using content analysis, as described by Elo and Kyngäs (2008). This study adheres to the standards for reporting qualitative research (SRQR) (O'Brien et al. 2014).

### 3.3 Sampling and Recruitment

The study was conducted in Sweden with inclusion criteria requiring current or prior employment as an NP in that country. Participants were recruited from regions spanning north to south, using a snowball sampling strategy. Initial contact was established with an NP known to the first author at a hospital in southern Sweden, who facilitated additional NP contacts through a national network. Subsequently, these NPs suggested further participants. Of the 16 individuals with NP experience invited to participate, 15 agreed. Each participant received an informational letter outlining the study, proposed interview dates and a consent form via email. Additionally, participants were informed that each interview was expected to take between 30 and 60 min.

### 3.4 Data Collection

The first author conducted 15 semi‐structured interviews during April and May 2022 using the Microsoft Teams digital platform, and participants granted consent for their interviews to be recorded and transcribed. The interviews lasted 20–57 min, with an average of 41 min. The interview guide used was centred on a key question: ‘Would you tell me about your experiences of the journey to becoming an NP and working in a new pioneering nursing role?’ As the interview progressed, more specific questions in the areas of education, competence and regulation were asked. The interview guide incorporated follow‐up and probing questions such as ‘Could you give some examples?’, ‘Do you mean?’ and ‘Why do you think?’ Each interview began with welcoming the participant and providing information about the study purpose and interview structure. Then, participants' background characteristics were obtained; they are presented in Table 1.

**TABLE 1:** Overview of the participants.

Number of participants	15 (all female)
Age	39–67 (mean 47)
Number of years as a registered nurse	11–39 (mean 21)
Number of years as a specialist nurse	5–29 (mean 14)
Number of years as a nurse practitioner	1–17 (mean 7)
Speciality area of clinical practice	Surgery care 9 Emergency care 2 Primary care 4

### 3.5 Data Analysis

The data underwent inductive content analysis at both manifest and latent levels, following the methodology outlined by Elo and Kyngäs (2008). The first author transcribed the interviews and iteratively reviewed the transcribed text to gain familiarity with and derive meanings from the data as a whole. The analysis, which was done manually commenced with open coding; initial notes were in the margins and subsequently transferred to a coding sheet. Codes were systematically organised into sub‐categories and subsequently into main categories in light of both differences and similarities. The coding process aimed not only to delineate variations and commonalities but also to encompass diverse aspects of the topic, facilitating a more comprehensive understanding (Elo and Kyngäs 2008). The analytical process was consistently discussed with the co‐authors, leading to new or changed codes and categories back and forward during the coding and categorising processes. Finally, consensus was achieved in the research team and four main categories emerged, each with two or three sub‐categories, forming the basis of the findings detailed in this report.

### 3.6 Ethical Considerations

The authors received an advisory opinion from the Swedish Ethical Review Board, diary number 2020‐02631, which determined that no formal ethical review was required under the Declaration of Helsinki. Adhering to the Declaration of Helsinki (World Medical Association 2013) as to participant anonymity, participants were informed both orally and in writing about the recording and verbatim transcription of the interviews. Written consent was obtained from all participants. They were also informed that participation was voluntary and that they had the right to withdraw at any time without providing further explanation. To further protect participant confidentiality, the organisational affiliations of the participants were not disclosed, given the limited number of NPs in Sweden.

### 3.7 Rigour

The trustworthiness of this study is evaluated using the criteria of credibility, dependability, confirmability and transferability (Elo and Kyngäs 2008). To enhance credibility, the research question was deemed to be most suitably addressed by individuals in semi‐structured interviews. This method enables the interviewer to tailor questions based on a given respondent's answers, thereby facilitating profound and unexpected insights. The study comprised 15 interviews, deemed sufficient to ensure data saturation (Polit and Beck 2020). To ensure content validity (Polit and Beck 2020) the initial interview served as a pilot to optimise the interview guide; no changes were made thereafter. The final interview reiterated patterns consistent with previous encounters, and the data comprehensively captured the diversity, depths and nuances of the research question.

To enhance transferability the recruitment strategy and the context in which the data were collected were described in detail. To improve dependability throughout the research process, the study adheres to the SRQR (O'Brien et al. 2014). To enable conformality and ensure that the interpretations of the findings in the analysis process are firmly rooted in the data and accurately represent the voice of the participants, a reflexive approach was maintained in the research team (Polit and Beck 2020). To avoid personal bias and enhance credibility, the coding process and the final categories underwent critical discussion within the team until consensus was reached (Elo et al. 2014). This process of investigator triangulation brought multiple perspectives that added depth and breadth to the analysis, ensuring the highest possible objectivity, given that all researchers were in the field of care science (Polit and Beck 2020). Additionally, the research team include members with expertise in conducting qualitative studies and content analysis. Moreover, the findings were enriched with authentic quotes extracted directly from the interviews, reinforcing the credibility grounded in the interviews (Polit and Beck 2020; Elo et al. 2014).

## 4 Findings

The findings are delineated across four categories, each comprising sub‐categories that cover the participants' transition into the NP role and their experiences in this pioneering nursing role (Table 2).

**TABLE 2:** Main categories and sub‐categories.

Progressing to the next level—motivators of and perspectives on NP education	Navigating loneliness and cultivating acceptance	Enhanced person‐centred care and supportive leadership	Lack of regulation and management influence autonomy and efficiency in the NP role
Addressing gaps in clinical career paths	Overcoming initial feelings of loneliness and dealing with doubts	Integrating nursing and medical competencies for holistic care	Different and changing levels of management support
Gradual competence development is crucial for mastering NP education	Acceptance comes through understanding but requires a lot of humility	The supervisor function led to safety and person‐centredness in the healthcare team	Dependence on physicians as a barrier to autonomy and efficiency in the NP role
Nationalising NP education to bridge local variants			Expanded prescribing rights would increase autonomy and effectiveness

### 4.1 Progressing to the Next Level—Motivators of and Perspectives on NP Education

This category describes how the NP role attracts experienced nurses, filling gaps in clinical career paths while allowing nurses to remain connected to patient care. The participants emphasised the gradual development of competence in NP education. Furthermore, participants raised concerns about variations in local NP programmes in the absence of a national standardised framework and advocated for structured progressions and physician collaboration.

#### 4.1.1 Addressing Gaps in Clinical Career Paths

The participants expressed a deep commitment to continuing personal and professional development. Their commitment to evolving in their roles as nurses while remaining engaged in direct patient care emerged as a central theme in their narratives. The NP role was as an attractive profession, offering a path for personal development while maintaining a close connection with patients. Moreover, the participants argued that well‐implemented NP roles played a crucial role in inspiring other nurses, thereby ensuring a steady supply of competence. In settings where the NP role was successfully integrated, the participants said that they were role models for less experienced colleagues. The NP role inspired other nurses to remain in clinical practice and over time train to become NPs; it could thus be important for the competence supply in the future. Furthermore, the participants highlighted the pressing need for well‐defined clinical career paths for experienced nurses. The absence of such paths, other than leaving clinical practice for management or academia, was considered detrimental to the healthcare competence supply. One participants expressed:

‘Because that was the way before [the NP role], if you want to get ahead as an experienced nurse then it was either to become a manager or start working at a university. This is to be developed further but still remains in the clinic. That was something that was very fun about this role that suits me very well.’ Interview 1

This deficiency was regarded as resulting in development‐focused nurses leaving clinical practice without opportunities for advancement. Consequently, the NP role was described as a feasible solution to effectively take advantage of the competence of experienced nurses by providing a compelling reason for them to remain in clinical practice. Participants explained that their intrinsic motivation derived from the NP role, where they could evolve as professionals without losing sight of their core identity as nurses.

‘But why I chose it [to become an NP] was probably that … you never become completely a scholar, but I still want to be developed in my role as a nurse, without getting further away from the patient.’ Interview 11

#### 4.1.2 Gradual Competence Development Is Crucial for Mastering NP Education

The participants underscored the consensus regarding the vital importance of establishing a robust foundation in nursing for NP education, building on their prior nursing competence. Participants reflected on the significance of ensuring a solid holistic approach even within medically oriented courses. Emphasising the need to prioritise holistic care over focusing solely on medical aspects, participants stressed the crucial role of nursing as a foundation to grasp the NP role. Participants pointed out that this nursing foundation facilitates gradual competence development, leading to a more profound and comprehensive understanding of both nursing and medicine. As one participant noted:

‘I feel that my nursing expertise has been strengthened by the fact that my medical theoretical and practical knowledge has been enhanced, and that I have both parts because it means that I can understand the overall picture in a completely different way.’ Interview 3

The participants argued that through this process they achieved the unique competence necessary to navigate the NP role and become prepared for the transition. Participants further argued that the main differences between their prior role as specialist nurses and the NP role lay in embracing a broader and deeper holistic perspective, which is based on their expanded nursing and medical skills. In the medical courses that are part of NP education, participants argued that they acquired skills encompassing proficiency in clinical assessments, pharmacology and the interpretation of various examination results. Participants regarded this as crucial to being an autonomous practitioner in line with the intention of the NP role.

‘We studied medical diagnostics and pharmacology at advanced level. But then there was also this tangible examination of patients, the systematic examination methodology that we had not received at all as nurses. You relate most to the anamnesis and to take vital parameters [as regular nurse], not these to feel, squeeze, listen and systematically examine the patients.’ Interview 14

The participants also shared valuable insights into their experiences as NP students during their internship periods and their gradual growth in the role. The collaboration between universities and healthcare institutions, coupled with physician engagement in the development process, emerged as critical factors. Physicians also served as supervisors during NP students' internships. The participants highlighted that having physicians as mentors enhanced collaborations, as the physicians became familiar with NP competencies and became comfortable working together. Some participants expressed that physicians eagerly included them in various educational sessions at the clinics. Due to their early involvement in the NP role development, physicians possessed an understanding of the specific knowledge required for NPs, enabling them to identify and address additional training needs. Most participants alternated theory and internship periods, which was an advantage because this iterative process equipped them with the competence they needed in the NP role. The opportunity to discuss a range of situations with physicians further developed their understanding.

In addition, participants emphasised the importance of requiring a background as a specialist nurse before entry into NP education. This prerequisite, participants argued, is crucial not only for defending the professional NP title but also to ensure a high level of competence. Some participants drew parallels with the structured progression in physicians' careers—they cannot go from intern to senior physician without taking a resident exam. The participants advocated a similar three‐step progression for NPs—registered nurse, specialist nurse and NP—citing the need for substantial prior competence before entering the NP role. Several participants underscored that NP education was notably advanced and comprehensive and observed that NP students lacking a specialist education encountered greater challenges in meeting course objectives because of insufficient experience upon which to build new knowledge: ‘I think it is dangerous if you as a newly graduated nurse almost immediately can step in and train for [NP] because you have to have something to relate everything to’ (Interview 6).

#### 4.1.3 Nationalising NP Education to Bridge Local Variants

Concerns were raised regarding the variations between local NP education programmes, which do not fully include the content necessary to match the international educational requirements. The participants expressed concern that some local education programmes do not require prior specialist education or include internships as part of the curriculum. Accordingly, worries were raised that locally developed education programmes might jeopardise the structured development of the NP role; this emphasises the need for a national framework to ensure a high‐quality education. In particular, participants highlighted the necessity of leveraging the structure of international programmes from countries where the NP role is already well established and integrated into educational systems. Several participants were actively involved in local education programmes, especially those who were working in surgical departments at university hospitals, where their involvement in training newly graduated NPs was anticipated. This engagement enabled them to offer valuable insights to the education based on their clinical experience within the NP role. Additionally, some participants were actively involved in national forums in which they shared their experiences and contributed to the ongoing development of the NP role. Participants advocated the inclusion of a master's level in NP education to ensure a high academic standard for the role and for navigating the professional NP role, emphasising evidence‐based knowledge of a high academic level for developing patient care. Some participants expressed concern that there could be a tendency to overlook the significance of such formal education in some healthcare settings, leading to the adoption of practices by which individuals take on expanded responsibilities without a formal NP education.

‘For example, in smaller hospitals, one might not recognize the importance of maintaining a high academic level, such as a master's degree [for the NP role]. Some may find it unnecessary, and in several areas, they have developed practice where individuals take on expanded responsibilities without a formal [NP] education. It is crucial for those of us who have a formal education to avoid the improvised variants spread across Sweden.’ Interview 5

### 4.2 Navigating Loneliness and Cultivating Acceptance

This category reveals the complexities of transitioning into the NP role, when participants encountered initial challenges of loneliness and self‐doubt. As pioneers, participants found solace in developing a sense of being between nursing and medicine. The journey demanded humility, persistence and a commitment to educating their healthcare teams about the NP role.

#### 4.2.1 Overcoming Initial Feelings of Loneliness and Dealing With Doubts

Participants encountered diverse challenges as newly graduated NPs. The initial phases involved a feeling of not belonging anywhere. Despite their foundational background in nursing, participants perceived that their connection to other nurses was not obvious; nor was it apparent in the medical profession. Consequently, they grappled with feelings of loneliness.

‘Very, very lonely… But you can see that there is a distance between me and them, even if it is the nurses or the physicians, you are in between. You do not really belong to any of the groups.’ Interview 9

While the initially difficulty of feeling like outsiders was challenging, it gradually became more manageable over time, as most participants found their place as a bridge between nursing and medicine. As they settled into this intermediary role, participants began to feel a sense of belonging to both the group of nurses and the group of physicians. Those with NP colleagues in their workplace had an even more favourable start, as the path had been paved by their predecessors, resulting in a strong support network and the absence of feelings of loneliness.

However, the transition to NP work was still challenging, and the participants reported that they had previously been experienced specialist nurses but felt like novices in the NP role. In the initial phase, it was crucial for NPs to concentrate on their new medical tasks, allowing them to practice and assert their position among other nurses. This focus was essential for breaking free from their previous nursing role and establishing their new role as NPs. Furthermore, the participants had doubts about whether they could cope with their new responsibilities. Many felt compelled to excel in all their duties to demonstrate their competence, especially regarding their medical assignments.

‘You have increased responsibilities and authorities, and then you have to demonstrate that you can handle them. So, of course there have been demands… To perform tasks that you traditionally do not undertake as a nurse without feeling confident in doing so, it was a challenge.’ Interview 9

#### 4.2.2 Acceptance Comes Through Understanding but Requires a Lot of Humility

Several participants highlighted the initial lack of acceptance of the NP role, attributing it to colleagues' unfamiliarity with NPs' responsibilities and mandate, including in their scope of practice and level of autonomy. The majority of participants found themselves continually having to explain their role to new team members who were unaware of what an NP was—an aspect they found frustrating. The extent of acceptance of the NP role varied; some experienced significant acceptance with well‐implemented roles aligning with the intended development. In workplaces where physicians had driven the development of the NP role, there was greater acceptance from medical professionals who actively supported NPs. Most physicians accepted the NP role, and even those who were initially resistant often changed their stance upon recognising its value.

All participants stressed the importance of colleagues being informed about the aim of the NP role and its scope of practice, delineating what NPs were and were not permitted to do. It was necessary to provide information not only to immediate colleagues in the hospital ward or healthcare centre but also to all the providers and managers encountered in daily work. There was unanimous agreement among participants regarding the need for additional clarification of the NP role.

‘I have described [it] for my colleagues many times… I said I am not a doctor, but I am the nurse who was here before, I am the nurse assistant that I have been before; no one can take that knowledge from me. I had just added an in‐depth competence.’ Interview 9

Moreover, obtaining acceptance from other nurses was often more challenging compared to physicians, with the degree of difficulty varying by workplace. Acceptance from newly graduated nurses was generally easier than more experienced ones. Some participants described changes in the behaviour of previous nursing colleagues upon their transition into the NP role, noting occasional instances of jealousy. While some participants continued to grapple with acceptance‐related issues to jealousy, they acknowledged that it required humility on their part as NPs to gain acceptance. Gradually, acceptance was achieved from both nurses and physicians. Building acceptance was notably easier among healthcare team members who had been part of their journey from NP student to NP graduate. Participants underscored the importance of providing information about the role to the healthcare team, as increased understanding led to recognition of the NPs' utility.

‘I know it was a nurse who asked me what I could do more than them. But when you had to show what you could do and that you have a higher level of competence and can take patients who would otherwise go to a doctor, then they realized that this [the NP role] is a very good thing that can make things easier for them and free up time and so on.’ Interview 10

### 4.3 Enhanced Person‐Centred Care and Supportive Leadership

This category describes how NPs facilitated the development of person‐centredness, both in direct patient care and within the healthcare team. Numerous participants derived substantial motivation from the expanded scope of their competence, enabling them to perform more comprehensive patient assessments and effectively bridging the divide between nursing and medicine. Furthermore, the participants argued that they play a crucial supervisory role in the NP position, serving as continuous support for the healthcare team.

#### 4.3.1 Integrating Nursing and Medical Competencies for Holistic Care

The expanded nursing and medical competencies in the NP role are vital in enhancing overall patient quality. Participants described how various professionals such as nurse assistants, nurses and physicians carry out their respective responsibilities independently. NPs, however, stand out by integrating both nursing and medical perspectives, thereby contributing to comprehensive patient management. Participants characterised themselves as seasoned nurses possessing profound medical expertise.

The participants conveyed that their expanded medical competence reinforced their nursing proficiency and, reciprocally, that their nursing skills enhanced their medical capabilities, fostering a more profound and holistic understanding of patient realities. However, participants clarified that the primary advantage in the NP role did not lie in undertaking new medical duties; rather, their strength resided in maintaining overall cohesion and advocating for the preferences of the patients.

‘Sometimes it feels like you have lost patient focus or what the patient really wants, and there are times I feel that I can be the one who sometimes must slow down or initiate actions in line with the patient's wishes.’ Interview 1

The participants highlighted their capacity to engage in conversations with patients that encompass both nursing and medical aspects, facilitating a more comprehensive understanding. Providing in‐depth explanations about the procedures during hospital admission and detailing post‐discharge medication changes were noted as particularly appreciated by patients. Participants further explained that they developed more comprehensive care plans than physicians and nurses working separately. Furthermore, participants attributed this to their holistic approach, which integrates both nursing and medical perspectives.

‘When I go to talk with a patient who should be discharged, it would not only be a conversation about their bile removal surgery. No, it becomes a whole that starts with nutrition, elimination, pain and so on. I take a comprehensive picture of what has happened and how to think further. No doctor does that; that is the truth.’ Interview 8

#### 4.3.2 The Supervisor Function Led to Safety and Person‐Centredness in the Healthcare Team

Overall, the participants highlighted the supervisory aspects of the NP role as crucial. They positioned themselves as a continuous presence in the healthcare setting, serving as a vital support to the entire healthcare team. Their independent role allowed them to provide ongoing assistance and supervision to team members, particularly when those people faced unfamiliar duties or uncertainties.

‘What I contribute is an overall focus on the patient, and I am a present, knowledgeable colleague whom you can turn to regardless of whether you are a nurse, an assistant nurse, a doctor at the beginning of your career or whatever you are. I am someone you can ask without its feeling scary; there are no stupid questions.’ Interview 1

In the context of hospital wards, the participants underscored the central role they played in mentoring newly graduated physicians. Their collaboration with interns was described as closely intertwined with their daily work, especially after medical rounds when senior physicians were engaged in surgical procedures. During these collaborative efforts, the NPs naturally assumed supervisory roles, providing trustworthy support to interns. Their guidance extended to decision‐making scenarios, including advising interns on appropriate actions and when the interns should involve a senior physician. Some participants detailed their experience in supervising new physicians. Initially, the Swedish Medical Association exhibited some reservation regarding NPs serving as mentors to newly graduated physicians on hospital wards. Concerns were raised that NPs might be inclined to assume the responsibilities of interns, potentially rendering them redundant. However, despite these concerns, these NPs ultimately received the annual mentorship prize for physicians interns.

### 4.4 Lack of Regulation and Management Influence Autonomy and Efficiency in the NP Role

From different perspectives, all participants underscored the pivotal role of management support or the lack thereof in the successful implementation of the NP role. In settings where management support was absent, the integration of the NP role faced challenges. Conversely, in environments with functional management support, the NP role operated effectively, and the healthcare team recognised the NPs' valuable contributions.

#### 4.4.1 Different and Changing Levels of Management Support

The levels of support from managers and physician varied among participants. While some experienced very strong support from their managers, some found their support to be merely sufficient, and others perceived a complete absence of support. Additionally, participants noted fluctuations in support over time, with some indicating that support was more robust during their NP education clinical practice, which occurred in their regular workplaces. Furthermore, some participants expressed that while management support was initially strong, it gradually waned. Notably, changes in managerial positions appeared to provoke significant disappointment among certain participants.

‘The head of care division and operations manager were replaced, so the vision that existed from the beginning from the organization's side, which certainly wasn't very concrete in the first place, but it was there in any case and was kind and strong. After they changed, there was no vision left because the new managers didn't have the same thought and ideas.’ Interview 7

The implementation of the NP role varied across different units, with success often linked to the involvement of physicians in management positions and the engagement of other physicians in role development. The daily work of NPs exhibited significant diversity based on the level of leadership support. Participants who expressed their desire to train as NPs to their managers encountered fewer conditions in implementing the role after graduation. While some participants initially experienced positive management attitudes, interest tended to decrease after graduation, leading to a lack of on‐going support for them. Participants highlighted challenges in role development without sustained management support, suggesting that NP effectiveness was hindered during such circumstances. The absence of healthcare education among certain managers was noted as a factor contributing to their difficulties in understanding the NP role. Despite the variability in daily work contingent on leadership support, NPs demonstrated resilience and determination in pursuing their new roles.

‘The implementation itself has been a big challenge that is not quite finished yet. But there's a long way left to implement a role in a setting where you have never heard about NPs before. So, I have said many times that I don't really give up until it's done. It's a struggle; there are new colleagues who come every week. I must repeat myself for everyone: who I am, what I do, what I can do and so on.’ Interview 9

In workplaces where management support was lacking, there were constant fears of being transferred from the role back to work as a regular nurse. This situation proved to be frustrating for some participants while others displayed greater acceptance. Participants working in hospital wards or emergency care conveyed the difficulty of reverting to regular nursing duties due to substantial differences in responsibilities. They noted that the dual role was challenging, with hospital ward NPs heavily involved in mentoring interns and performing tasks that encompass both nursing and medical perspectives. The transition to regular nursing duties meant assuming responsibility for all traditional nursing tasks, leaving inadequate time for NP duties. This shift from basic nursing to comprehensive medical duties adversely affected efficiency. By contrast, NPs in primary care were better positioned to leverage their expanded competence and conduct more comprehensive assessments during patients interactions. This capability contributed to increased efficiency along the care chain, allowing NPs in primary care to effectively deploy their enhanced skills.

#### 4.4.2 Dependence on Physicians as a Barrier to Autonomy and Efficiency in the NP Role

Participants outlined several barriers in their daily work that impeded their autonomy and contributed to inefficiencies in the care chain. All the participants were given expanded responsibilities to perform tasks that had previously been reserved for physicians. This included ordering blood tests, and other diagnostic procedures, such as writing X‐ray referrals, prescribing medications and discharging patients from the hospital. However, the circumstances under which participants performed these tasks varied, which in turn influenced the level of independence practice they experienced in their NP role. Key obstacles included limited prescribing capabilities and digital care systems designed exclusively for physician use. The practical aspects of the NPs' daily work significantly influenced participants efficiency, leading to frustration because they consistently relied on physicians, creating barriers to autonomy. Participants unanimously advocated a protected professional title to enhance autonomy and efficiency in the care chain.

Despite encountering barriers, participants devised strategies to navigate challenges and effectively fulfil their medical duties. However, participants faced hindrances in systems that restricted usage to physicians, forcing NPs to rely on physicians' logins even when delegated certain responsibilities. This included instances where NPs had to use physicians' names to order blood samples, a practice with which participants were uncomfortable, despite formal delegation. Successful implementation of this approach was noted to build trust and confidence between physicians and NPs. Additionally, autonomy in some workplaces was contingent on the preferences of the physician of the day, introducing variability in NPs' ability to function in their roles.

‘It depended on which doctor was on duty if I could work as an NP or not; some days it was not even a concept because the doctor did not listen to my assessment… If you knew the doctor, it was fine to work as NP, but otherwise there was no idea [of that].’ Interview 14

The participants highlight the necessity to address legal ambiguities surrounding the NP role to provide clarity on its mandate. However, contrasting views were expressed, with some participants feeling that they already possessed the necessary mandate and authority to independently carry out many duties effectively. The collaborative decision‐making process within the team was emphasised, allowing NPs to autonomously implement decisions that had been made collectively. While most workplaces had a job description that proved crucial support for NPs, there is a recognised need for a national description to further advance the NP role. Accordingly, participants strongly emphasised the necessity of a protected professional title to regulate their mandate and authority, fostering increased autonomy: ‘But I absolutely think it is important to have a protected professional title, partly to get a national description of what an NP is allowed to do, but also to get a national coordination [of the NP role]’ (Interview 7).

#### 4.4.3 Expanded Prescribing Rights Would Increase Autonomy and Effectiveness

Participants, particularly those in primary and emergency care, emphasised the significance of having the right to prescribe medication. Hospital ward NPs also acknowledged its importance, stating that it streamlined their work and improved the efficiency of the care chain. However, many participants did not currently have that right, which specialist nurses could obtain after completing a 15‐credit course (though participants were in the process of completing that course). Despite lacking prescription rights, participants highlighted that their extensive nursing experience led them to approach medication decisions with a comprehensive assessment of a patient's overall situation. Nonetheless, those with the legal right to prescribe medication as nurses described it as a facilitator in their daily work. Opinions varied among participants regarding the scope of prescription rights, with some suggesting alignment with intern capabilities, while others believed that a modest expansion of the medical list would enhance their autonomy.

‘But I have to say that you can get very far without the prescription right, as much of that should not actually be more than what you can advise or prescribe as a specialist nurse because it is far from everyone who needs antibiotics. Surprisingly, you need help with just a few prescriptions.’ Interview 11

Most participants had a designated list of medications they were authorised to prescribe, granted through a general delegation for the ward's areas of care. Despite this, several participants asserted that the significance of the NP role was not diminished without this right, as they contributed substantially in their other capacities. The absence of prescription rights was seldom an issue in well‐implemented NP roles where physicians had accepted and trusted NPs, willingly writing the needed prescriptions. However, concerns were raised by some participants who felt that relying on delegation or physician prescriptions was not ideal. Their apprehension centred on jurisdictional matters, potentially creating unclear lines of responsibility between physicians and NPs. This uncertainty made NPs uneasy and could limit their ability to fully exercise their autonomous roles: ‘Let's say I make an incorrect assessment, and it becomes a liability case, and a doctor has prescribed antibiotics, and the patient dies; who is responsible for that? Is it me, or is it the doctor?’ (Interview 14).

## 5 Discussion

The findings offer valuable insights into the experiences of working in the NP role in the Swedish context. The findings not only illuminate the advantages and barriers associated with NP education and practice but also shed light on the motivations that drive experienced nurses to pursue NP roles. These insights provide a deeper understanding of the complexities and nuances of the NP profession in Sweden's healthcare system. The findings align with previous research conducted in that country. Despite more than a decade having passed since the previous evaluations of local initiatives, NPs still face challenges with role clarity, acceptance and regulation issues (Altersved et al. 2011; Bergman et al. 2013; Lindblad et al. 2010). However, the consistency between past evaluations and our findings underscores the enduring potential of the NP role to enhance patient care and improve the effectiveness of healthcare teams (Altersved et al. 2011; Bergman et al. 2013; Lindblad et al. 2010). Notably, both our study and previous research indicate positive patient outcomes stemming from the NP's pivotal role in delivering person‐centred care and nurturing an overall culture of person‐centredness within healthcare teams. For example, the findings suggest that the NP's central position as a bridge between nursing and medicine contributes to a comprehensive approach to patients. Additionally, NPs enhance safety and increase competence among team members, including newly graduated physicians. The participants' commitment to mentorship, collaboration and providing support to the entire healthcare team demonstrates the significance of NPs in facilitating a cohesive and effective healthcare environment. This finding accords with prior international research (Henni et al. 2018; Morilla‐Herrera et al. 2016) highlighting the effectiveness of the NP role in healthcare teams. For instance, Henni et al. (2018) suggest that NPs' contributions have several dimensions, including both direct and indirect effects on patient care quality; that is in line with our findings. The direct effect stems from NPs' expanded competencies, enabling them to grasp a wider spectrum of patients' medical, physical and social situations and leading to a holistic approach to care. Additionally, consistent with our findings, Henni et al. (2018) describe the indirect effects on patient care quality resulting from NPs' consulting and tutoring functions within healthcare teams. Similar insights into the direct and indirect effects of NPs are also reflected in a prior Swedish study highlighting NPs' roles in delivering person‐centred and holistic care with high continuity, as well as their support of the healthcare team (Ljungbeck, Carlson, and Sjögren‐Forss 2022).

Furthermore, the findings reflected how NPs consistently prioritise nursing activity over medical treatment, illuminating their commitment to nursing principles. This viewpoint contradicts the perception that the NP role is intended to replace physicians, an issue highlighted in previous studies (Andregård and Jangland 2015; Thompson and McNamara 2022). Instead, our findings underscore the complementary relationship between NPs and physicians in settings where the NP role has been effectively implemented. Moreover, NPs clearly position themselves as experienced nurses with extended nursing and medical skills rather than as aspiring to become solely medical professionals. This distinction could serve as a valuable benchmark for ongoing dialogues by affirming that the NP role aims to complement rather than supplant physicians. For example, Rickards and Hamilton (2020) describe how the effectiveness of the NP role transcends NPs being merely ‘mini physicians’ who are cost‐effective. Instead, NPs provide healthcare from a distinct perspective rooted in their historical identity as nurses. Those authors reject comparisons with physicians and advocate for inclusion as a new role within multidisciplinary teams working collaboratively to meet patients' needs.

The findings also uncovered significant challenges faced by NPs, including initial feelings of loneliness and obstacles in attaining autonomy in patient care. While the sense of loneliness tends to decrease over time, the reliance on physicians continues to impede NPs' effectiveness, primarily due to unclear regulations. Limitations in autonomy stem from practical constraints and vague mandates, resulting in dependence on physicians for various tasks and negatively affecting the efficiency of care delivery. Despite the instances of significant autonomy experienced by some NPs, the findings suggest that autonomy is closely tied to supportive leadership and the commitment of physicians to developing the NP role. Previous studies (Andregård and Jangland 2015; Thompson and McNamara 2022) corroborate these difficulties and underscore the necessity of supportive management and clear regulations to enhance NP autonomy. These challenges are not solely attributed to unclear regulation of the NP role but also to the entrenched hierarchical structures within healthcare systems.

However, in line with our findings, Andregård and Jangland (2015) propose that managers in healthcare organisations should be careful when introducing the NP role within healthcare teams, in light of challenges common to organisational changes. Our study, supported by prior research (Dehennin et al. 2023; Thompson and McNamara 2022), indicates that providing clear information on the NP role and the intentions for implementing it is a viable approach. Nevertheless, to mitigate NPs' dependency on supportive leadership and physician engagement in practice, it is imperative to ensure a national framework to bolster the introduction of the NP role at the local level (Ljungbeck, Carlson, and Sjögren‐Forss 2023). We contend that the diversity of localised solutions, where the design of mandates relies solely on individual managers or physicians at a given care unit, is an inadequate approach because it creates unequal conditions for different NPs to work independently, even if they possess the same level of education. Instead, as the findings underscore, there is a critical need for a national framework to effectively introduce NPs into Swedish healthcare teams. Previous studies have reached the same conclusion: the absence of regulation around the NP role and the lack of supportive management create varying conditions for and limitations on NPs, leading to confusion in healthcare teams and impeding the overall effectiveness of the NP role (Lockwood et al. 2022; Torrens et al. 2020).

Furthermore, our findings suggest that the new medical mandates incorporated into the NP role require regulation at the national level. While they indicate that NPs can operate effectively without the ability to prescribe medications, formalising this capability could significantly enhance efficiency along the care chain. The findings reveal that NPs often prioritise nursing activities before considering medication, making prescription rights a valuable addition to their scope of practice. Such rights are especially crucial in emergency and primary care settings, where immediate access to a physician may not always be feasible. A prior study (Maier 2019) illustrates the diversity in NP prescription rights across European countries; while some NPs possess the authority to prescribe all types of medications independently, others have more limited privileges, often requiring physician supervision. In comparison, English speaking countries like Australia, the United Kingdom, Canada and Ireland where the NP role has been established for many years, prescription rights are generally more regulated and standardised than in European countries. In these English‐speaking nations, most NPs are authorised to prescribe a broad range of drugs, including antibiotics, antivirals, anticoagulants and cholesterol‐lowering drugs, without physician oversight. With the exception of Canada, all of these countries also permit NPs to prescribe controlled substances, such as narcotics and strong pain relievers. In Canada, however, NPs can only prescribe controlled drugs in the context of palliative care (Delamaire and Lafortune 2010). In light of this variability, we argue that it has become imperative to establish a coherent framework for NP prescriptions in Sweden. A suitable approach aligned with our findings could involve augmenting the existing legal prescription form in the NP role to include additional medications. This adjustment would enhance NP autonomy and streamline the care chain.

Another crucial finding in this study underscores the importance of defining admission criteria for NP education programmes. Participants stressed prior specialist knowledge and experience as prerequisites for entry. Concerns were raised regarding the admission policies of some local NP programmes, which may not require prior specialist education. This aligns with prior research advocating a uniform national framework to ensure consistent standards and competence levels across NP education programmes (MacKay et al. 2018; Schallmo et al. 2019). In light of our findings, we posit that advancing the NP role in Sweden necessitates the establishment of a standardised national framework that would serve to mitigate concerns surrounding the inconsistent nature of admission policies and foster uniformity in NP education standards.

## 6 Strengths and Limitations

One strength of this study lies in its data collection method, which included participants from diverse regions spanning north to south in Sweden. This approach contributes to a comprehensive understanding of NPs' experiences and offers a genuinely nationwide perspective. Given the limited research focused on Sweden, the findings have the potential to enhance our understanding of the pioneering NP role in that country, benefiting decision‐makers and other stakeholders involved in its development. The study's trustworthiness is reinforced by its alignment with prior international research, which identifies similar experiences in the NP role. Limitations stem from the use of digital platforms for interviews. While digital methods may reduce participant inhibitions and enhance anonymity, they may also compromise non‐verbal cues and interview depth compared to face‐to face interactions (Williams et al. 2012). However, considering the target group, this aspect was considered of secondary importance, as prioritising the breadth of collective NP experiences from a national perspective was deemed paramount for the aim of the study. Another factor that could both strengthen and weaken the study, potentially impacting the trustworthiness of the findings, is the predominance of female participants. This demographic composition, however, likely mirrors the reality of the nursing field, where woman make up significant majority, with approximately 90% of nurses worldwide being female (Kearns and Mahon 2021).

## 7 Conclusion

The findings of the present study offer a comprehensive overview of the varied experiences of working in the pioneering NP role in Sweden. They underscore the impact of an unregulated role and reveal a range of challenges, including reliance on physicians, limited autonomy, insufficient management support, initial feelings of loneliness and a lack of clarity. Despite these challenges, the findings highlight the significant value that NPs may bring to Swedish healthcare settings. Their contributions indicate that NPs can promote continuity of care, enhance healthcare team safety, optimise care chain efficiency, improve patient outcomes and deliver person‐centred care. This recognition of the NP role in enhancing the quality and effectiveness of healthcare services is a crucial aspect of this study. Moreover, the insights from this study are pertinent not only to the Swedish context but also to the international experience. Understanding the realities of working in the NP role can facilitate decision‐makers and foster best practices in the development process.

## Conflicts of Interest

The authors declare no conflicts of interest.

## Peer Review

The peer review history for this article is available at https://www.webofscience.com/api/gateway/wos/peer‐review/10.1111/jan.16560.

## Supporting information


**Data S1** SRQR_Checklist.

## Data Availability

Data available on request from the authors.
